# Changes in blood rheological properties and biochemical markers after participation in the XTERRA Poland triathlon competition

**DOI:** 10.1038/s41598-022-07240-1

**Published:** 2022-03-01

**Authors:** Aneta Teległów, Jakub Marchewka, Łukasz Tota, Dawid Mucha, Bartłomiej Ptaszek, Robert Makuch, Dariusz Mucha

**Affiliations:** 1 Institute of Clinical Rehabilitation, University of Physical Education in Krakow, Krakow, Poland; 2 Institute of Biomedical Sciences, University of Physical Education in Krakow, Krakow, Poland; 3 Institute of Health Sciences, Podhale State College of Applied Science in Nowy Targ, Nowy Targ, Poland; 4 Institute of Applied Sciences, University of Physical Education in Krakow, Krakow, Poland; 5 Kazimierz Pulaski University of Technology and Humanities in Radom, Radom, Poland

**Keywords:** Biochemistry, Immunology, Physiology

## Abstract

The importance of physical activity in preventing chronic cardiovascular and metabolic diseases and the role of exercise as an adjunct therapy are widely recognized. Triathlon is a typically endurance discipline. Prolonged and intensive exercise is known to cause changes in blood rheological properties and biochemical markers; sometimes athletes participating in strenuous competitions need medical attention. To understand the phenomena occurring in the body in such situations, we decided to study participants’ biomarkers after the XTERRA Poland 2017 triathlon competition. The study involved 10 triathletes. The XTERRA Poland 2017 event comprised 1500-m swimming, 36-km cycling, and 10-km mountain running. Blood samples were collected 2 days before, immediately after, and 16 h after the competition. Immediately after the race, white blood cells count, platelets, and uric acid levels were significantly (*P* < 0.001) increased; haematocrit, Na^+^, Cl^–^, and IgA were decreased. On the following day, Na^+^, Cl^–^, and C-reactive protein levels were significantly (*P* < 0.001) increased; white blood cells count, red blood cells count, haemoglobin, haematocrit, mean corpuscular volume, platelets, IgG, and IgA were decreased. Assessing rheological parameters such as erythrocyte deformability and aggregation is useful for monitoring adverse effects of intensive and exhaustive exercise. The study illustrates the change in blood rheological properties and biochemical markers after intensive physical effort. Despite these differences, the indicators were within the reference range for the general population, which may demonstrate normal body function in the studied triathletes.

## Introduction

The importance of physical activity in preventing chronic cardiovascular and metabolic diseases and the role of exercise as an adjunct therapy are widely recognized. It has been demonstrated in epidemiological studies that increased blood viscosity, haematocrit, plasma viscosity, red cell aggregation, and leukocyte count are related to several cardiovascular diseases^1,2^. However, prolonged and intensive exercise is known to cause changes in blood rheological properties and biochemical markers, and sometimes athletes participating in strenuous competitions need medical attention. What is specific about triathlon is that it combines 3 different sports (swimming, cycling, and running)^3^. This constitutes a huge challenge for the human body, necessitating great strength, endurance, and resistance to fatigue, pain, and stress. The athletes are required to be perfect in each of these 3 disciplines, which means that they have to start preparing early enough. The comprehensive training prepares triathletes for a strenuous effort in all aspects. Very often participants receive physicians’ assistance to monitor vital signs such as systolic and diastolic blood pressure, heart rate, stroke volume, lung minute ventilation. Training, diet, and other activities are individually tailored to each athlete during the preparation period. Intensive physical exercise induces numerous changes and responses in the human body. Blood rheological parameters considered together with morphological and biochemical blood indicators provide valuable information about the health status. As indicated by El-Sayed^4^, blood flow properties are altered in athletes; special attention should be focused here on plasma viscosity, fibrinogen concentration, and haematocrit. Mairbäurl^5^ implies that younger red cells are more deformable, which leads to improved tissue oxygen supply during exercise. Numerous studies referring to blood indicators in triathletes have confirmed morphological, rheological, and biochemical changes^6–10^.

The aim of this paper was to assess the impact of training- and competition-related factors in the XTERRA Poland triathlon event on changes in morphological, rheological, and biochemical blood indicators: before triathlon, immediately after triathlon, and 16 h after triathlon. The available literature includes few studies related to this objective. In our research, we aimed to provide a broader perspective of analyses with regard to numerous biochemical indicators.

The following research question was posed: What is the range and direction of the changes in morphological, rheological, and biochemical blood indicators in the group of investigated triathletes before triathlon, immediately after triathlon, and after a 16-h post-triathlon rest?

Despite the risk of losing health benefits, triathlon is a considerable challenge for both athletes and amateurs. What are the capabilities of the human body? It is known that regular physical exercise improves metabolism, which is essential for preventing cardiovascular diseases and reducing the risk of mortality.

## Material and methods

### Study group

The study group comprised 10 males aged 32.8 ± 3.1 years, members of the Active Side of Life Association (Krakow, Poland), who regularly practised triathlon. The mean training experience in the study group was 10 ± 4.2 years. The participants’ average sports level corresponded to the I and II sports class (national level competitors).

### The XTERRA Poland 2017 competition

The athletes took part in the XTERRA Poland 2017 triathlon event (1,500-m swimming, 36-km cycling, and 10-km mountain running), which constituted the XTERRA world championship qualifications. The competition was held on August 13, 2017.

The mean (range) temperature during the sports event was 31.2 ± 3.1 °C (25–33 °C), with a relative humidity of 69 ± 8% (60–71%) and the dew point of 21 ± 3 °C (18–25 °C). The swimming part was performed in a water reservoir with a water temperature of 20 ± 1 °C. At this stage of the race, all participants were equipped with neoprene swimsuits. When cycling, the competitors used bicycles with carbon or aluminium frames. Before the competition, the participants were not instructed on the amount of liquids or energy products to consume during the race in order to avoid the influence of these factors on the final result. However, after the competition, all athletes were asked to estimate the amount of fluid consumed at each nutrition point. The average fluid intake during the race equalled 0.7 ± 0.3 l of water and 1.5 ± 0.5 l of isotonic drinks.

### Blood sample collection

The triathletes’ blood samples were collected 2 days before the competition (on August 11, 2017), on the triathlon day immediately after all races (on August 13, 2017), and 16 h after triathlon (on August 14, 2017). Approximately 10 ml of blood was collected each time into EDTAK2 VACUETTE tubes and clot tubes. The fasting blood collection procedure was performed by a qualified nurse before triathlon in the morning (measurement 1) and 16 h after triathlon (measurement 3) in the Laboratory of Blood Physiology of the University of Physical Education in Krakow and immediately after the race (measurement 2) at the competition site, i.e. Zalew Zakrzówek (artificial water reservoir in Krakow, Poland). For the first blood measurement, the athletes were expected to be well-rested, after a minimum of one day free of any training load, properly hydrated, and having eaten a light meal at least 8 h before the blood sampling. The collected material was analysed in the Laboratory of Blood Physiology of the University of Physical Education in Krakow and in the Department of Analytics and Clinical Biochemistry of the Krakow Oncology Centre.

### Somatic indicator assessment (Table 1)

**Table 1:** Selected somatic indicators in the examined competitors.

Group	BM (kg)	BH (cm)	LBM (kg)	FM (kg)	F% (%)
Competitors	81.1 ± 3.0	181.7 ± 4.1	70.0 ± 2.9	11.1 ± 3.0	13.7 ± 2.6

*BM* body mass, *BH* body height, *LBM* lean body mass, *FM* fat mass, *F%* percentage of fat tissue.

The anthropometric measurements 2 days before the competition involved body height, body mass, fat mass, and lean body mass. Body mass and composition were determined with the use of a Jawon Medical, model IOI 353 (Korea) body composition analyser. Body height was evaluated with a Martin (USA) anthropometer, with a measurement accuracy of 1 mm.

### Blood parameter measurements

The measurement of basic haematological indicators was performed in a HORIBA ABX Micros 60 blood analyser with the impedance method (Micros 60, HORIBA ABX, Montpellier, France). Blood morphology indicators were presented as preliminary results in a study by Teległów et al.^11^; here, they have been extended to provide plasma volume changes, as well as rheological and biochemical indicators. Considering dehydration during exercise, specific changes in plasma volume were taken into account in accordance with the van Beaumont^12^ formula:

$$ \% \Delta {\text{PV}} = \left[ {100/\left( {100{-}{\text{HCT}}1} \right)} \right] \times \left[ {100\left( {{\text{HCT}}1{-}{\text{HCT}}2} \right)/{\text{HCT}}2} \right] $$

where %ΔPV—percentage change in plasma volume, HCT1—haematocrit before exercise, HCT2—haematocrit after exercise.

As for blood rheology, the aggregation and deformability of red blood cells were tested with the Laser-assisted Optical Rotational Cell Analyser (LORCA) (Mechatronics, the Netherlands) and the method by Hardeman et al.^13^.

To measure electrolytes, a Cobas device (Roche, Germany) was used with an ion-selective electrode module to quantify potassium, sodium, and chloride ions. The sodium and potassium electrodes are based on natural carriers, and the chloride electrode is based on an ion exchanger. The following electrolytes were analysed: Na^+^ [mmol/l]—concentration of sodium ions, K^+^ [mmol/l]—concentration of potassium ions, Cl^–^ [mmol/l]—concentration of chloride ions.

The renal and liver profile was measured by using a Roche/Hitachi Cobas c 311 analyser. The indicators were determined with the colorimetric method by using reagent kits and a Cobas c 311 analyser (Roche Diagnostics). For each sample, the device automatically calculates the analytical activity of the given substance. In the renal profile, urea [mmol/l]^14^, creatinine [mmol/l]^15^, and uric acid [μmol/l]^16^ were measured. In the liver profile, total bilirubin [μmol/l]^17^, AST [U/l]—aspartate transaminase^18,19^, ALT [U/l]—alanine transaminase^20,21^, and GGT [U/l]—gamma-glutamyltransferase^22^ were evaluated. These indicators were determined with the colorimetric method by using reagent kits and a Cobas c 311 analyser (Roche Diagnostics).

A Dade Behring device in the BN ProSpec system was applied to assess the diagnostic indicators for determining immunoglobulins. The immunonephelometric method served to investigate immunoglobulins (IgG, IgA, IgM) in human serum with reagents designed for in-vitro diagnostics^23^. C-reactive protein (CRP; an acute phase protein) concentration was evaluated with the immunonephelometric method^24,25^, by using reagent kits and a BN ProSpec nephelometer (Siemens Health). Protein electrophoresis was performed in a Cobas c 311/511 analyser in Roche/Hitachi systems^26^.

The concentrations of albumin and the other fractions (alpha-1-globulin, alpha-2-globulin, beta-1-globulin, beta-2-globulin, gamma-globulin) were calculated on the basis of the total protein concentration and the percentage of electrophoretic fraction, determined in the analysis of electropherograms, obtained after separation of serum proteins by capillary electrophoresis (Minicap, Sebia).

### Statistical analysis

The data were presented as means and standard deviations or medians and quartiles I and III, depending on the normal distribution assessment. The normality of distributions was examined with the Shapiro–Wilk test. Mauchly’s test served to verify the sphericity assumption. The pre- and post-triathlon variables were compared by using the analysis of variance (ANOVA) for repeated measures and, if the assumptions were not met, with the Friedman ANOVA by ranks test. Post-hoc evaluation employed the Tukey/Bonferroni test or the post-hoc Friedman ANOVA test, constituting a part of the Statistica medical package. The percentage change in plasma volume was calculated with one-way ANOVA. The level of significance of α = 0.05 was assumed. The analyses were performed with the Statistica 12 software (StatSoft^®^, USA).

### Ethical approval

This research was approved by the Ethical Committee at the Regional Medical Chamber in Krakow (approval No.: 17/KBL/OIL/2015) and conformed to the principles of the Declaration of Helsinki.

### Consent to participate

Written informed consent was obtained from all participants included in the study.

### Consent for publication

The authors affirm that human research participants provided informed consent for this publication.

## Results

Measurement 1 was performed 54 h before triathlon, measurement 2—immediately after triathlon, measurement 3–16 h after triathlon. The mean values and standard deviations of the obtained results are presented in the tables below.

### Blood morphology (Table 2)

**Table 2:** Mean values (± SD) of blood morphology indicators in the 3 performed measurements (M).

Parameter	Before triathlon (M1)	Immediately after triathlon (M2)	16 h after triathlon (M3)	*P* ANOVA	Post hoc		
					M1/2	M2/3	M1/3
WBC (10^9^/l)	6.25 ± 2.16	14.53 ± 3.73	7.50 ± 1.66	*	*	*	0.634
RBC (10^12^/l)	5.18 ± 0.23	4.89 ± 0.20	4.40 ± 0.18	*	*	*	*
HGB (g/l)	15.45 ± 0.81	14.54 ± 1.01	12.96 ± 0.87	*	*	*	*
HCT (l/l)	44.60 ± 1.89	41.71 ± 3.03	37.70 ± 2.25	*	*	*	*
MCV (fl)	86.21 ± 3.18	85.16 ± 3.50	85.70 ± 3.23	*	*	0.104	0.328
MCH (fmol)	29.67 ± 1.11	29.71 ± 1.11	29.47 ± 1.51	0.849	*P* ≥ 0.05	*P* ≥ 0.05	*P* ≥ 0.05
MCHC (mmol/l)	34.44 ± 0.91	34.89 ± 0.91	34.42 ± 1.63	0.723	*P* ≥ 0.05	*P* ≥ 0.05	*P* ≥ 0.05
PLT (10^9^/l)	248.50 ± 44.64	269.63 ± 54.94	214.50 ± 43.81	*	*	*	*

*Significant difference (*P* < 0.05).

*WBC* white blood cells count, *RBC* red blood cells count, *HGB* haemoglobin, *HCT* haematocrit, *MCV* mean corpuscular volume, *MCH* mean corpuscular haemoglobin, *MCHC* mean corpuscular haemoglobin concentration, *PLT* platelets.

When comparing the status before triathlon and immediately after triathlon, an increase in white blood cells count (WBC) by as much as 56% was observed. The platelet count (PLT) rose by 7.8%. The result analysis also revealed reductions in red blood cells count (RBC) by 5.6%, haemoglobin (HGB) by 5.9%, haematocrit (HCT) by 6.5%, and mean corpuscular volume (MCV) by 1.2%.

As for the values reported immediately after triathlon versus 16 h after triathlon, WBC decreased by 48.8%, PLT by 20.5%. RBC was again reduced by 5.6%. The analysis also demonstrated a decrease in HGB by 10.9% and in HCT by 9.6%. The remaining indicators did not change statistically significantly.

With regard to the comparison of results obtained before triathlon versus 16 h after triathlon, lower values of most investigated indicators were observed. RBC decreased by 15.1%, HGB by 16.1%, HCT by 15.5%, PLT by 13.7%. The analysis did not reveal statistically significant changes in the remaining indicators.

### Plasma volume (Table 3)

**Table 3:** Percentage changes in plasma volume in the 3 performed measurements.

Before triathlon versus immediately after triathlon	Immediately after triathlon versus 16 h after triathlon	Before triathlon versus 16 h after triathlon
− 10.94 ± 8.10	− 19.41 ± 5.85	− 31.20 ± 14.59
Between the ‘before triathlon versus immediately after triathlon’ and ‘immediately after triathlon versus 16 h after triathlon’ changes: *P* + 0.2703		
Between the ‘before triathlon versus immediately after triathlon’ and ‘before triathlon versus 16 h after triathlon’ changes: *P* = 0.0027*		
Between the ‘immediately after triathlon versus 16 h after triathlon’ and ‘before triathlon versus 16 h after triathlon’ changes: *P* = 0.0905		

*Significant difference (*P* = 0.0020).

During a long-lasting effort in an environment with increased temperature and relative humidity (temperature: 31.2 ± 3.1 °C, relative humidity: 69 ± 8%), a percentage increase in plasma volume was observed by 10.94 ± 8.10% (before triathlon vs. immediately after triathlon). The increase equalled 19.41 ± 5.85% when comparing the status immediately after triathlon and 16 h after triathlon, and 31.20 ± 14.59% between before triathlon and 16 h after triathlon.

### Red blood cells: elongation index (Table 4, Fig. 1) and aggregation indices (Table 5)

**Table 4:** Mean values (± SD) of the elongation index at various levels of shear stress in the 3 performed measurements (M).

Shear stress (Pa)	Before triathlon (M1)	Immediately after triathlon (M2)	16 h after triathlon (M3)	*P* ANOVA	Post hoc		
					M1/2	M2/3	M1/3
0.30	0.06 ± 0.02	0.07 ± 0.02	0.04 ± 0.03	0.0837			
0.58	0.08 ± 0.01	0.07 ± 0.02	0.06 ± 0.03	0.164			
1.13	0.12 ± 0.01	0.10 ± 0.02	0.08 ± 0.04	0.134			
2.19	0.19 ± 0.02	0.15 ± 0.03	0.12 ± 0.07	*	0.030	0.347	*
4.24	0.26 ± 0.03	0.21 ± 0.04	0.17 ± 0.09	*	0.010	0.286	*
8.23	0.33 ± 0.04	0.26 ± 0.05	0.21 ± 0.10	*	0.002	0.271	*
15.98	0.39 ± 0.04	0.30 ± 0.05	0.25 ± 0.13	*	0.002	0.271	*
31.03	0.45 ± 0.04	0.35 ± 0.06	0.28 ± 0.14	*	0.005	0.183	*
60.30	0.46 ± 0.06	0.38 ± 0.06	0.32 ± 0.14	*	0.040	0.313	*

*Significant difference (*P* < 0.05).

**Figure 1:**
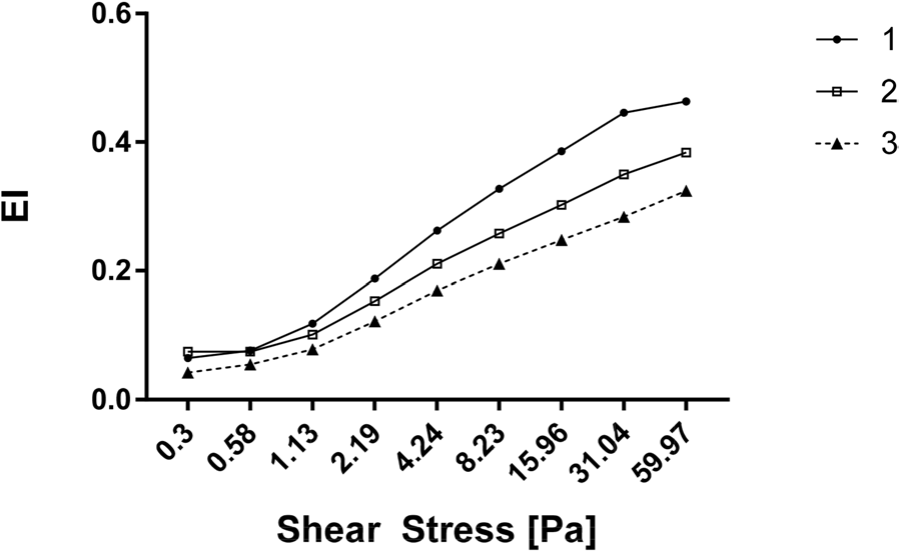
Red blood cells: elongation index (EI) depending on shear stress in the performed measurements (1, 2, 3).

**Table 5:** Mean values (± SD) of the aggregation index (AI), aggregation half-time (T1/2), and amplitude of aggregation (AMP) in the 3 performed measurements (M).

Parameter	Before triathlon (M1)	Immediately after triathlon (M2)	16 h after triathlon (M3)	*P* ANOVA
AI (%)	19.29 ± 1.98	17.07 ± 3.38	18.02 ± 5.05	0.763
T1/2 (s)	2.47 ± 0.98	3.50 ± 1.88	3.26 ± 1.89	0.069
AMP (au)	60.92 ± 8.32	59.61 ± 17.96	65.40 ± 22.20	0.418

When comparing the status before triathlon and immediately after triathlon, no statistically significant differences were found for the elongation index (EI) at the shear stress of 0.30–60.30 Pa or for the aggregation index (AI), amplitude of aggregation (AMP), aggregation half-time (T1/2).

As for the values reported immediately after triathlon versus 16 h after triathlon, no statistically significant differences were observed for EI at the shear stress of 0.30–60.30 Pa, and there were no statistically significant differences for the other indices: AI, AMP, or T1/2.

With regard to the comparison of results obtained before triathlon versus 16 h after triathlon, when analysing the mean EI values, statistically significant differences were found at the shear stress of 2.19–60.30 Pa. No statistically significant differences were revealed for AI, AMP, or T1/2.

### Blood biochemical indicators: electrolytes (Table 6)

**Table 6:** Mean values (± SD) of electrolyte concentrations in the 3 performed measurements (M).

Parameter	Before triathlon (M1)	Immediately after triathlon (M2)	16 h after triathlon (M3)	*P* ANOVA	Post hoc		
					M1/2	M2/3	M1/3
Na^+^ (mmol/l)	141.40 ± 1.71	139.88 ± 1.55	142.10 ± 0.99	*	*	*	0.541
K^+^ (mmol/l)	4.65 ± 0.23	4.56 ± 0.36	4.41 ± 0.31	*	0.513	0.171	0.022
Cl^–^ (mmol/l)	105.28 ± 1.39	101.45 ± 2.89	104.69 ± 1.93	*	*	*	0.892

*Significant difference (*P* < 0.05).

When comparing the status before triathlon and immediately after triathlon, a statistically significant decrease was found in the levels of Na^+^ (by 1.08%) and Cl^–^ (by 2.62%) after triathlon. The levels of K^+^ did not present any statistically significant changes.

As for the values reported immediately after triathlon versus 16 h after triathlon, there was a statistically significant increase of Na^+^ (by 1.6%) and Cl^–^ (by 3.2%) levels. The remaining changes did not present any statistical significance.

With regard to the comparison of results obtained before triathlon versus 16 h after triathlon, a statistically significant decrease of the level of K^+^ (by 5.2%) was revealed. The remaining changes did not present any statistical significance.

### Renal function tests (Table 7)

**Table 7:** Mean values (± SD) or medians (IQR) of renal function indicators in the 3 performed measurements (M).

Parameter	Before triathlon (M1)	Immediately after triathlon (M2)	16 h after triathlon (M3)	*P* ANOVA	Post hoc		
					M1/2	M2/3	M1/3
Urea (mmol/l)	6.12 ± 1.62	7.35 ± 1.76	6.95 ± 0.94	0.081			
Creatinine (mmol/l)	89.79 ± 12.69	108.51 ± 17.63	92.13 ± 14.34	*	*	*	0.999
Uric acid (μmol/l)	307.27 ± 50.19	436.08 ± 78.76	382.18 ± 52.69	*	*	*	*
eGFR (ml/min/1.73 m^2^)	77.0 (73.0–90.0)	67.0 (59.5–77.5)	83.5 (65.0–90.0)	*	*	*	0.972

*Significant difference (*P* < 0.05).

*IQR* interquartile range, *eGFR* estimated glomerular filtration rate.

When comparing the status before triathlon and immediately after triathlon, it was observed that intensive physical effort (running, cycling, swimming) had increased blood creatinine concentration [mmol/l] in triathletes by 17.25% and raised uric acid concentration [μmol/l] by 29.54%. For the estimated glomerular filtration rate (eGFR) [ml/min/1.73 m^2^], a decrease was revealed by 12.98%. No statistically significant changes were observed for urea [mmol/l].

As for the values reported immediately after triathlon versus 16 h after triathlon, a statistically significant decrease in blood creatinine concentration [mmol/l] was found by 15.1%, and there was a decrease in uric acid concentration [μmol/l] by 12.13%; however, eGFR [ml/min/1.73 m^2^] increased by 19.76%. No statistically significant changes were observed for urea [mmol/l].

With regard to the comparison of results obtained before triathlon versus 16 h after triathlon, a statistically significant increase in uric acid concentration [μmol/l] by 19.6% was determined. No statistically significant changes were observed for urea [mmol/l], creatinine [mmol/l], or eGFR [ml/min/1.73 m^2^].

### Liver function tests (Table 8)

**Table 8:** Medians (IQR) of liver function indicators in the 3 performed measurements (M).

Parameter	Before triathlon (M1)	Immediately after triathlon (M2)	16 h after triathlon (M3)	*P* ANOVA	Post hoc		
					M1/2	M2/3	M1/3
Total bilirubin (mol/l)	9.65 (7.50–14.10)	14.65 (10.00–17.20)	19.05 (11.50–21.80)	*	0.782	0.053	*
AST (U/l)	26.95 (22.90–34.20)	30.35 (28.55–44.05)	37.75 (31.90–67.20)	*	0.327	0.120	*
ALT (U/l)	23.20 (20.80–29.20)	22.70 (18.30–25.40)	24.90 (18.50–29.00)	0.419			
GGT (U/l)	21.25 (14.90–24.50)	18.40 (12.85–22.00)	18.70 (13.90–24.20)	*	0.803	0.091	*

*Significant difference (*P* < 0.05).

*IQR* interquartile range, *AST* aspartate transaminase, *ALT* alanine transaminase, *GGT* gamma-glutamyltransferase.

When comparing the status before triathlon and immediately after triathlon, no statistically significant changes were found for total bilirubin [μmol/l], AST [U/l], ALT [U/l], or GGT [U/l].

As for the values reported immediately after triathlon versus 16 h after triathlon, no statistically significant changes were observed for total bilirubin [μmol/l], AST [U/l], ALT [U/l], or GGT [U/l].

With regard to the comparison of results obtained before triathlon versus 16 h after triathlon, there was a statistically significant increase in total bilirubin [μmol/l] by 49.35%, an increase in AST [U/l] by 28.61%, and a decrease in GGT [U/l] by 12%. No statistically significant changes were revealed for ALT [U/l].

### Immunoglobulins (IgG, IgA, IgM), protein electrophoresis, C-reactive protein (Table 9)

**Table 9:** Mean values (± SD) or medians (IQR) of selected serum protein parameters in the 3 performed measurements (M).

Parameter	Before triathlon (M1)	Immediately after triathlon (M2)	16 h after triathlon (M3)	*P* ANOVA	M1/2	M2/3	M1/3
Total protein (g/l)	71.09 ± 2.50	71.40 ± 4.36	67.31 ± 2.63	*	0.910	*	*
Albumin (g/l)	44.73 ± 1.62	44.98 ± 2.30	42.59 ± 1.22	*	0.989	*	*
Alpha-1-globulin (g/l)	2.40 ± 0.33	2.34 ± 0.30	2.36 ± 0.27	1.000			
Alpha-2-globulin (g/l)	5.73 ± 0.31	5.21 ± 0.65	5.43 ± 0.52	*	*	0.881	*
Beta-1-globulin (g/l)	4.20 ± 0.55	4.45 ± 0.50	3. 90 ± 0.42	*	0.855	*	*
Beta-2-globulin (g/l)	3.41 ± 0.53	3.38 ± 0.67	3.17 ± 0.54	*	0.240	0.240	*
Gamma-globulin (g/l)	10.69 ± 1.67	11.08 ± 1.92	9.91 ± 1.64	*	0.825	*	*
A/G (g/l)	1.70 ± 0.14	1.71 ± 0.15	1.74 ± 0.16	0.070			
IgG (g/l)	12.71 ± 1.72	13.09 ± 2.01	11.89 ± 1.78	*	0.769	*	*
IgA (g/l)	2.58 ± 0.83	2.41 ± 0.75	2.07 ± 0.67	*	< 0.001	*	*
IgM (g/l)	0.93 ± 0.28	0.95 ± 0.28	0.84 ± 0.27	*	0.108	*	*
CRP (mg/l)	0.71 (0.32–2.15)	0.57 (0.19–1.44)	7.05 (2.29–9.01)	*	0.999	0.001	< 0.001

*Significant difference (*P* < 0.05).

*IQR* interquartile range, *A/G* albumin/globulin ratio, *CRP* C-reactive protein.

When comparing the status before triathlon and immediately after triathlon, a decrease was found in alfa-2-globulin [g/l] by 9.07% and in IgA [g/l] by 6.6%. No statistically significant changes were observed for total protein [g/l], albumin [g/l], alpha-1-globulin [g/l], beta-1-globulin [g/l], beta-2-globulin [g/l], gamma-globulin [g/l], albumin/globulin ratio (A/G), IgG [g/l], IgM [g/l], or CRP [mg/l].

As for the values reported immediately after triathlon versus 16 h after triathlon, the observed decrease in total protein [g/l] by 5.73%, decrease in albumin [g/l] by 5.31%, decrease in beta-1-globulin [g/l] by 12.36%, decrease in gamma-globulin [g/l] by 9.93%, decrease in IgG [g/l] by 9.17%, decrease in IgM [g/l] by 11.58%, and increase in CRP [mg/l] by 1136.84% were statistically significant.

With regard to the comparison of results obtained before triathlon versus 16 h after triathlon, the observed decrease in total protein [g/l] by 5.32%, decrease in albumin [g/l] by 4.78%, decrease in alpha-2-globulin [g/l] by 6.81%, decrease in beta-1-globulin [g/l] by 7.14%, decrease in beta-2-globulin [g/l] by 7.04%, decrease in gamma-globulin [g/l] by 7.3%, decrease in IgG [g/l] by 5.74%, decrease in IgA [g/l] by 19.77%, decrease in IgM [g/l] by 9.68%, and increase in CRP [mg/l] by 892.96% were statistically significant. No statistically significant changes were noted for alpha-1-globulin [g/l] or A/G.

## Discussion

The presented study is among the few to analyse changes in morphological, rheological, and biochemical blood indicators in athletes training triathlon. The aim of our research was to analyse a number of indicators whose monitoring may contribute to a more comprehensive optimization of the training process in endurance athletes. Understanding the ‘battleground’ inherent in triathlon and other endurance events can allow for a better insight into the body adaptation processes during and around the start period.

Among the studies published so far, there are few reports that address triathletes’ blood morphology. Most morphological indicators have been investigated in runners or cyclists. Blood morphological indicators in triathletes well characterize the actual extent and direction of effort changes and allow to diagnose transient adaptive effects. No extreme results of particular biochemical, rheological, or morphological indicators were observed in the athletes at the different stages of the study. The findings confirm that intensive physical effort during the triathlon resulted in an increase in leukocyte and platelet counts; however, 16 h after the competition, their values were similar to those at baseline. This phenomenon was most likely caused by intensive exercise, stress, or even the consumption of a large meal before the race. The increase in WBC is most often explained by a decrease in plasma volume during increased activity^27^. Researchers suggest that WBC may also be influenced by consuming a large meal before training, by intensive effort itself, or even by stress before or during the competition, manifesting itself in increased adrenaline, noradrenaline, or cortisol levels. This has been confirmed in a study by Tota et al.^7^. According to Maron et al.^28^, an increase in the number of leukocytes in the body supports the principle that training improves athletes’ immune function. However, a 4-year study of Spanish triathletes revealed that WBC remained within normal limits both before and during competition^29^. Yet, in an Australian Institute of Sport study, 16% of triathletes exhibited neutropenia and 5% presented with monocytopenia^30^. Philip and Bermon^30^ indicate that individuals with neutropenia are generally more susceptible to bacterial infections, which can occur after inadequate treatment or minor skin injuries. The reason for neutropenia remains unclear. It may be due to exercise-induced apoptosis of neutrophils and, consequently, their shorter life duration.

One possible reason for the increase in PLT after intensive training may be the loss of plasma in the blood after such a strenuous exercise, and the reduction in PLT in triathletes (within normal limits) after the competition constitutes a return to baseline values. Intensive effort increases the activity of platelets and clotting factors, which ensures blood fluidity^31^.

The analysis of the red blood cell system revealed that RBC, HGB, and HCT decreased both immediately after the competition and 16 h later; this resulted from enhanced post-exercise haemolysis or the risk of anaemia. According to Banfi et al.^32^, HGB and HCT are reduced when more intensive training is implemented throughout the season. In different sports disciplines, the decrease in HGB ranges from 3 to 8% during the competition season, while the reticulocyte range varies from 5 to 21%. As indicated by Mairbäurl^5^, exercise can decrease red blood cell mass as a result of intravascular haemolysis of senescent red blood cells, which occurs with the mechanical rupture when the cells pass through capillaries in contracting muscles. In turn, Brun et al.^33^ implied that a lower level of HCT in competitive athletes compared with non-athletes resulted in higher aerobic capacity, whereas a too high level of HCT (> 44.6%) led to overtraining, anaemia, and increased blood viscosity. Our results are supported by a study by Rietjens et al.^34^, who also observed that numerous elite (presumably Olympic distance) triathletes exhibited RBC values below the lower limit of normal. These findings indicate that the phenomenon was caused by post-exercise haemolysis, which involves a decrease in the number of erythrocytes accompanied by haemoglobin passage into the blood plasma. Erythrocyte destruction is due to erythrocyte forcing through capillaries and to such factors as increased acidity, pressure, body temperature, muscle contractions causing pressure on blood vessels, oxidative stress, red blood cell volume changes. Another cause of post-exercise haemolysis is low blood sugar level (hypoglycaemia). This is a temporary phenomenon in athletes, resulting from insufficient carbohydrate intake prior to intensive physical effort. The decrease in RBC in the studied triathletes may also be related to excessive iron loss^35,36^. Currently, there are no established levels of iron requirements for athletes of specific sports disciplines. In training individuals, iron stores are excreted mainly with urine and, to a lesser extent, with sweat. One should also consider dietary errors, as well as mechanical injuries. The latter, as a result of regular and prolonged hitting of the foot against the ground, destroy red blood cells in the blood vessels of the lower limbs (foot-strike haemolysis)^37^. Some have postulated that haemolysis may be related to other, non-traumatic factors^38,39^. Researchers suggest that the decrease in HGB may be due to haemolysis, which is the mechanism of erythrocyte destruction during and after physical activity^40–43^. Furthermore, the HGB reduction may be caused by haemodilution, which is associated with endurance training and results from increased plasma volume in athletes^44^. Malcovati et al.^45^ demonstrated that a reduction in HCT could develop with intensive physical activity immediately after a long break from training.

With regard to the change in plasma volume, during a long-lasting effort in an environment with increased temperature and relative humidity (temperature: 31.2 ± 3.1 °C, relative humidity: 69 ± 8%), an increase in plasma volume was observed by 10.94 ± 8.10% (before triathlon vs. immediately after triathlon). The increase equalled 19.41 ± 5.85% when comparing the status immediately after triathlon and 16 h after triathlon, and 31.20 ± 14.59% between before triathlon and 16 h after triathlon. The increase in plasma volume in triathletes may be related to hormonal changes that underlie the decrease in HCT.

Physical activity leads to numerous changes in blood rheological properties. Rheological parameters involve those assessing erythrocyte deformability and aggregation. All are useful for monitoring the particularly undesirable short- and long-term effects of practising extreme sports such as triathlon. The main factors affecting blood flow (blood rheology) include erythrocyte deformability and aggregation, plasma viscosity, and haematocrit index.

Dehydration and the high oxygen demand exert a very strong influence, which can lead to red blood cells remodelling and transformation of their adaptability. Erythrocytes contain 62% of water; most of the water is responsible for their deformability, oxygen transport, and other properties. Free water constitutes approximately 25%. During short-term exercise, the amount of water does not change or slightly decreases, while the amount of free water increases. During training, free water decreases and bound water increases in the erythrocyte, with unchanged erythrocyte volume. Bound water affects erythrocyte deformability^46^. Long-term dehydration destabilizes the erythrocyte cell membrane and increases its rigidity. Therefore, adequate hydration during and after physical effort should be strongly promoted in order to prevent excessive blood viscosity. Blood thinning during training can be beneficial to delivering oxygen to muscles by reducing resistance to flow. In addition, increased plasma volume can contribute to water storage in the body and help compensate for dehydration.

In our study, statistically significant changes in red blood cell deformability were observed only when comparing the status before triathlon versus 16 h after triathlon. When analysing the mean EI values, statistically significant differences were found at the shear stress of 2.19–60.30 Pa. Reduced red blood cell deformability is an important determinant of impaired perfusion, increased blood viscosity, ischaemia, and capillary obstruction. It can also reduce oxygenation of organs and impair their physiological functions. Deterioration of erythrocyte elastic properties may be caused by dehydration through increased oncotic pressure of colloid inside the vessels^2^.

No statistically significant differences were revealed for indicators of deformability and red blood cell aggregation: EI, AI, AMP, T1/2; this phenomenon is most likely due to the degree of the athletes’ training.

Immediately after triathlon, we found a statistically significant decrease in Na^+^ (by 1.08%) and Cl^–^ (by 2.62%) levels. The slight lowering of Na^+^ and Cl^–^ concentrations most probably results from the participants’ mean fluid consumption during the race, which equalled 0.7 ± 0.3 l of water and 1.5 ± 0.5 l of isotonic drinks. When comparing the status immediately after triathlon versus 16 h after triathlon, we observed a statistically significant increase of Na^+^ (by 1.6%) and in Cl^–^ (by 3.2%) levels. For the comparison between before triathlon and 16 h after triathlon, a statistically significant decrease of K^+^ (by 5.2%) was revealed. The remaining changes did not present any statistical significance.

Fluctuations (within normal limits) in the concentrations of Na^+^, K^+^, and Cl^–^ in the competitors immediately after triathlon or 16 h after triathlon depend on the type and amount of consumed fluids. Hiller^47^ points at dehydration as a common phenomenon, and at exercise-related hyponatraemia as the predominant electrolyte disorder at Ironman Hawaii. Ad libitum fluid intake is the best way to maintain plasma Na^+^ concentration in triathletes. In a Half Ironman, however, oral salt supplementation improved performance^48^, effectively lessening body mass loss and increasing serum electrolyte concentration^48^.

Renal function expressed by eGFR was lower in triathletes by 12.98% immediately after the event and increased by 19.76% after 16 h. During physical effort, fluid shifts occur between the intravascular and interstitial spaces; fluid loss with sweat is also observed. Moreover, there are changes in hormone concentrations, e.g. increases in adrenaline, glucagon, cortisol, or adrenocorticotropic hormone. This, in turn, may involve changes in creatinine concentrations. Creatinine is derived from creatine metabolism and its turnover in healthy individuals is constant owing to the total muscle mass that corresponds to almost 100% of creatine storage in the body^49^. Creatinine is commonly used as an indicator of kidney function. Intensive physical effort (running, cycling, swimming) increased blood creatinine concentration in triathletes by 17.25% and raised uric acid concentration by 29.54%, but 16 h after triathlon, blood creatinine concentration was decreased by 15.1% and uric acid by 12.13%. With intensive physical effort, skeletal muscle hypoxia may occur, which is manifested, among others, by raised creatinine concentration. To summarise the differences for the renal profile indicators, according to Puggina et al.^50^, these effects are probably due to the exercise-induced modifications in the glomerular membrane and endocrine variables such as antidiuretic hormone, catecholamines, and aldosterone.

There were no statistically significant changes in the levels of hepatocyte intracellular enzymes such as AST or ALT, total bilirubin, or GGT in triathletes immediately after the competition or 16 h later. Although physical effort causes an increase in liver enzyme levels, which quickly return to normal after a period of rest, this was not demonstrated in our study. When comparing the status before triathlon versus 16 h after triathlon, we found a statistically significant increase in total bilirubin (by 49.35%), as well as an increase in AST (by 28.61%) and decrease in GGT (by 12%).

The main factors increasing bilirubin in athletes are haemolysis and the subsequent haemoglobin catabolism^51^. The accelerated break-down of red blood cells mainly results from mechanical phenomena (marching haemolysis, damage caused by muscle work, or red blood cells squeezing through capillaries) and from the destructive effects of free radicals^52–55^. Total bilirubin is among the parameters used to assess liver function, and elevated levels may indicate liver dysfunction or damage. Other parameters applied to evaluate liver function include alkaline phosphatase, ALT, and GGT. Despite the observed differences, liver enzyme activity was within the accepted reference range for the general population, which may be indicative of normal liver function in the investigated triathletes.

When analysing the statistically significant changes in immunoglobulins (IgG, IgA, IgM) and protein electrophoresis, a decreasing tendency can be observed. A decrease in the mean values of total protein, albumin, globulin, and IgG, IgA, and IgM antibodies may reflect an inhibitory effect of intensive physical effort on the immune system. It is most likely that increased concentrations of such hormones as adrenaline and cortisol inhibit immune system activity during and after competition. In previous years, Gleeson et al.^56^ found that high-intensity exercise caused a negative effect on the immune system by inhibiting IgA secretion, while moderate exercise could positively impact on the immune system. They also observed that immediately after very strenuous training, IgA and IgM levels decreased, but usually returned to normal after 24 h. Finally, the authors concluded that low levels of IgM and IgA might adversely affect the immune system. Gleeson et al.^57^ maintain that IgA is associated with upper respiratory tract protection, which would support the hypothesis that intensive physical effort reduces immunity^56,57^. Libicz et al.^58^ revealed decreased levels of total protein and IgA in saliva among 8 professional triathletes in the 2001 French Iron Tour event. IgA concentration decreased by 51.9% after the competition compared with the fasting level before the race. On this basis, the researchers concluded that regular and intensive exercise had a negative impact on the athletes’ immune system.

CRP concentration increased by 1136.84% when comparing the status immediately after triathlon and 16 h after triathlon, and by 892.96% for before triathlon versus 16 h after triathlon. The increased CRP levels in the investigated athletes prove that such intensive exercise damages muscle structures. These results, together with those achieved by Bach et al.^59^, lead to a conclusion that damage to structures occurs during exercise, but increased CRP levels are evident as late as after 24 h.

## Conclusions

The obtained results are crucial for high performance of athletes during triathlon competitions. Monitoring changes in particular morphological, rheological, and biochemical blood indicators should contribute to diagnosing overload conditions in endurance athletes.

Despite the observed differences, many indicator levels reported in the triathletes remain within the accepted reference ranges for the general population, which may be indicative of normal body functions in the investigated participants. However, further research should focus on an in-depth understanding of the mechanisms of adaptation to physical effort of varying volume and intensity in individuals with different levels of physical capacity. The extent to which the observed changes constitute an adaptation to stress induced by physical effort should be considered.

To sum up, changes in the morphological, rheological, and biochemical blood indicators were found as a result of intensive effort during triathlon. The crucial ones, which should first be addressed in the periodization of the training process among endurance athletes, are the increase in CRP concentration by 1136.84%, increase in creatinine concentration by 17.25%, increase in uric acid concentration by 29.54%, and decrease in IgA concentration by 51.9% after the competition. Further research is needed to clarify whether these changes can be considered adaptations to this type of exercise, which combines swimming, cycling, and running.

## Data Availability

All data generated or analysed during the study are included in this article.
